# (Acetyl­acetonato-κ^2^ *O*,*O*′)carbon­yl[tris­(naphthalen-1-yl)phosphane-κ*P*]rhodium(I) acetone hemisolvate

**DOI:** 10.1107/S1600536812008148

**Published:** 2012-03-10

**Authors:** Hezron Ogutu, Reinout Meijboom

**Affiliations:** a Research Centre for Synthesis and Catalysis, Department of Chemistry, University of Johannesburg, PO Box 524, Auckland Park, 2006 Johannesburg, South Africa

## Abstract

The title compound, [Rh(C_5_H_7_O_2_)(C_30_H_21_P)(CO)]·0.5C_3_H_6_O, has two different complex molecules in the asymmetric unit, with the Rh^I^ atoms in slightly distorted square-planar coordination environments. The molecules are packed as two monomeric mol­ecules with one acetone solvent mol­ecule sitting at the centre.

## Related literature

For related literature on the catalytic activities of rhodium phosphine adducts, see: Carraz *et al.* (2000 ▶); Moloy & Wegman (1989 ▶). For related complexes, see: Bonati & Wilkinson (1964 ▶); Brink *et al.* (2007 ▶); Leipoldt *et al.* (1978 ▶); Janse van Rensburg *et al.* (2006 ▶).
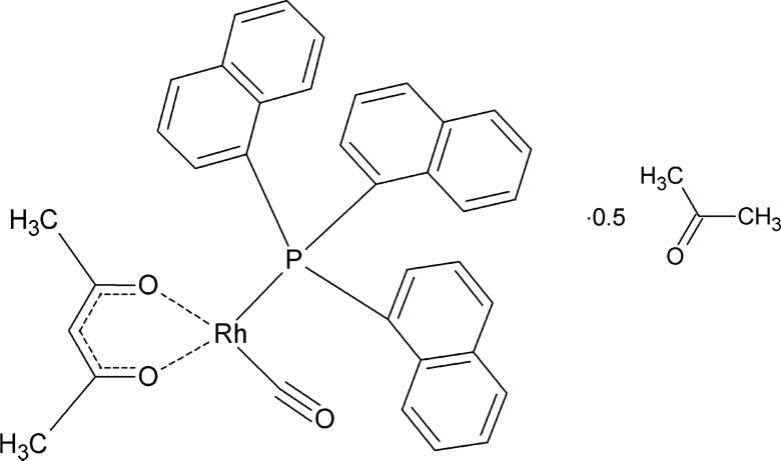


## Experimental

### 

#### Crystal data

[Rh(C_5_H_7_O_2_)(C_30_H_21_P)(CO)]·0.5C_3_H_6_O
*M* *_r_* = 671.51
Monoclinic, 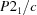

*a* = 19.8780 (5) Å
*b* = 16.9350 (5) Å
*c* = 18.8060 (4) Å
β = 102.916 (1)°
*V* = 6170.6 (3) Å^3^
*Z* = 8
Cu *K*α radiation
μ = 5.27 mm^−1^
*T* = 173 K
0.15 × 0.09 × 0.04 mm

#### Data collection

Bruker APEXII CCD diffractometer
Absorption correction: multi-scan (*SADABS*; Bruker, 2007 ▶) *T*_min_ = 0.505, *T*_max_ = 0.817
105322 measured reflections
10278 independent reflections
9730 reflections with *I* > 2σ(*I*)
*R*_int_ = 0.043

#### Refinement

*R*[*F* ^2^ > 2σ(*F* ^2^)] = 0.028
*wR*(*F* ^2^) = 0.068
*S* = 1.08
10278 reflections
745 parameters
240 restraints
H-atom parameters constrained
Δρ_max_ = 1.56 e Å^−3^
Δρ_min_ = −0.59 e Å^−3^

### 

Data collection: *APEX2* (Bruker, 2007 ▶); cell refinement: *SAINT-Plus* (Bruker, 2007 ▶); data reduction: *SAINT-Plus*; program(s) used to solve structure: *SHELXS97* (Sheldrick, 2008 ▶); program(s) used to refine structure: *SHELXL97* (Sheldrick, 2008 ▶); molecular graphics: *SHELXTL* (Sheldrick, 2008 ▶); software used to prepare material for publication: *SHELXTL*.

## Supplementary Material

Crystal structure: contains datablock(s) global, I. DOI: 10.1107/S1600536812008148/hg5181sup1.cif

Structure factors: contains datablock(s) I. DOI: 10.1107/S1600536812008148/hg5181Isup2.hkl

Additional supplementary materials:  crystallographic information; 3D view; checkCIF report

## Figures and Tables

**Fig. 1.**
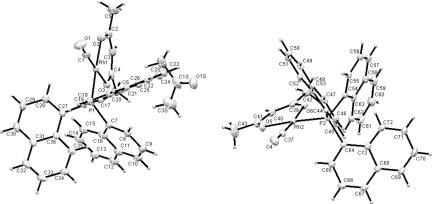
Molecular structure of the title compound showing 50% probability displacement ellipsoids.

## supplementary crystallographic information

### Comment

Acetylacetonate has two O-donor atoms with equivalent σ-electron donor capabilities. The high symmetry of dicarbonyl(acetylacetonate)rhodium(I) complexes promotes easy carbonyl displacement of either carbonyl group with a variety of phosphines, phosphites and arsines. (Bonati & Wilkinson, 1964). This work is part of an ongoing investigation aimed at determing the steric effects induced by various phosphine ligands on a rhodium(I) metal centre. Previous work illustrating the catalytic importance of the rhodium(I) square- planar moieties has been conducted on rhodium mono- and di-phosphine complexes containing the symmetrical bidentate ligand, acac (acac = acetylacetonate) (Moloy & Wegman, 1989). Symmetrical di-phosphine ligands result in the production of acetaldehyde, whereas unsymmetrical di-phosphine ligands are more stable and efficient catalysts for the carbonylation of methanol to acetic acid (Carraz *et al.*, 2000).

In the title compound, the Rh lies at the base of acetylacetonato ring. The coordination polyhedron around the Rh atom shows a slightly distorted square-planar arrangement. A larger *trans* influence of the phosphine ligand with respect to the carbonyl ligand is indicated by the longer Rh—O2 2.0633 (17) Å) bond compared to Rh—O3 2.0380 (17) Å) bond which is *trans* to the carbonyl ligand. The steric demand of the phosphine is indicated by the smaller O3—Rh1—P1 angle, 92.78 (5)°), compared to the carbonyl ligand (O2—Rh1—C1 = 90.07 (9)°). Similar geometries have been observed for related complexes [Brink *et al.* (2007); Leipoldt *et al.* (1978); Janse van Rensburg *et al.* (2006)].

Spectroscopic characteristics of the current compound are similar to that reported previously by Brink *et al.* (2007), and we refer at Brink *et al.* (2007) for additional discussion on the spectroscopy of these types of compounds.

### Experimental

A solution of [Rh(acac)(CO)_2_] (25.8 mg, 0.1 mmol) in acetone (5 cm^3^) was slowly added to a solution of [P(C_10_H_7_)] (41.2 mg, 0.1 mmol) in acetone (5 cm^3^) at room temperature, the mixture was then stirred for 10 min. Slow evaporation of the solvent afforded the title compound as a yellow crystalline solid.Spectroscopic analysis: ^31^P{H} NMR (CDCl_3_, 161.99 MHz, p.p.m.):46.42 [d, ^1^J(Rh—P) = 179.81 Hz]; IR ν(CO): 1971.2 cm^-1^; (CD_2_Cl_2_) ν(CO): 1982.8 cm^-1^.

### Refinement

The aromatic, methine, and methyl H atoms were placed in geometrically idealized positions (C—H = 0.95–0.98) and constrained to ride on their parent atoms, with *U*_iso_(H) = 1.2*U*_eq_(C) for aromatic and methine H atoms, and *U*_iso_(H) = 1.5*U*_eq_(C) for methyl H atoms respectively. Methyl torsion angles were refined from electron density.

### Crystal data

**Table:**

[Rh(C_5_H_7_O_2_)(C_30_H_21_P)(CO)]·0.5C_3_H_6_O	*F*(000) = 2752
*M**_r_* = 671.51	*D*_x_ = 1.446 Mg m^−^^3^
Monoclinic, *P*2_1_/*c*	Cu *K*α radiation, λ = 1.54184 Å
Hall symbol: -P 2ybc	Cell parameters from 9789 reflections
*a* = 19.8780 (5) Å	θ = 3.9–64.9°
*b* = 16.9350 (5) Å	µ = 5.27 mm^−^^1^
*c* = 18.8060 (4) Å	*T* = 173 K
β = 102.916 (1)°	Plate, yellow
*V* = 6170.6 (3) Å^3^	0.15 × 0.09 × 0.04 mm
*Z* = 8	

### Data collection

**Table:**

Bruker APEXII CCD diffractometer	9730 reflections with *I* > 2σ(*I*)
Graphite monochromator	*R*_int_ = 0.043
φ and ω scans	θ_max_ = 64.9°, θ_min_ = 3.9°
Absorption correction: multi-scan (*SADABS*; Bruker, 2007)	*h* = −23→23
*T*_min_ = 0.505, *T*_max_ = 0.817	*k* = −19→19
105322 measured reflections	*l* = −21→16
10278 independent reflections	

### Refinement

**Table:**

Refinement on *F*^2^	Primary atom site location: structure-invariant direct methods
Least-squares matrix: full	Secondary atom site location: difference Fourier map
*R*[*F*^2^ > 2σ(*F*^2^)] = 0.028	Hydrogen site location: inferred from neighbouring sites
*wR*(*F*^2^) = 0.068	H-atom parameters constrained
*S* = 1.08	*w* = 1/[σ^2^(*F*_o_^2^) + (0.0274*P*)^2^ + 9.4181*P*] where *P* = (*F*_o_^2^ + 2*F*_c_^2^)/3
10278 reflections	(Δ/σ)_max_ = 0.003
745 parameters	Δρ_max_ = 1.56 e Å^−^^3^
240 restraints	Δρ_min_ = −0.59 e Å^−^^3^

### Special details

**Table:**

Geometry. All e.s.d.'s (except the e.s.d. in the dihedral angle between two l.s. planes) are estimated using the full covariance matrix. The cell e.s.d.'s are taken into account individually in the estimation of e.s.d.'s in distances, angles and torsion angles; correlations between e.s.d.'s in cell parameters are only used when they are defined by crystal symmetry. An approximate (isotropic) treatment of cell e.s.d.'s is used for estimating e.s.d.'s involving l.s. planes.
Refinement. Refinement of *F*^2^ against ALL reflections. The weighted *R*-factor *wR* and goodness of fit *S* are based on *F*^2^, conventional *R*-factors *R* are based on *F*, with *F* set to zero for negative *F*^2^. The threshold expression of *F*^2^ > σ(*F*^2^) is used only for calculating *R*-factors(gt) *etc*. and is not relevant to the choice of reflections for refinement. *R*-factors based on *F*^2^ are statistically about twice as large as those based on *F*, and *R*- factors based on ALL data will be even larger.

### Fractional atomic coordinates and isotropic or equivalent isotropic displacement parameters (Å^2^)

**Table:**

	*x*	*y*	*z*	*U*_iso_*/*U*_eq_	
Rh2	0.534136 (9)	0.265488 (10)	0.656237 (9)	0.01388 (5)	
Rh1	0.900894 (9)	0.212742 (10)	0.226138 (10)	0.01600 (6)	
P2	0.44700 (3)	0.20502 (3)	0.69398 (3)	0.01280 (12)	
P1	0.97479 (3)	0.14922 (3)	0.31614 (3)	0.01495 (13)	
O2	0.82855 (9)	0.26594 (10)	0.14433 (10)	0.0245 (4)	
O1	0.89175 (12)	0.06892 (12)	0.13498 (11)	0.0399 (5)	
O4	0.61587 (9)	0.11875 (11)	0.68514 (11)	0.0290 (4)	
O1S	0.69700 (11)	0.22018 (13)	0.40521 (13)	0.0419 (5)	
O3	0.90469 (9)	0.31368 (10)	0.28592 (9)	0.0206 (4)	
O5	0.61765 (9)	0.32029 (10)	0.62888 (10)	0.0244 (4)	
O6	0.47819 (9)	0.36556 (9)	0.62588 (9)	0.0182 (4)	
C11	1.00411 (14)	0.30098 (16)	0.49516 (16)	0.0283 (2)	
C37	0.58290 (13)	0.17534 (15)	0.67555 (13)	0.0192 (5)	
C50	0.41440 (12)	0.01493 (15)	0.45279 (13)	0.0202 (5)	
H50	0.4079	−0.004	0.4042	0.024*	
C52	0.43376 (12)	0.12415 (14)	0.53699 (13)	0.0160 (5)	
H52	0.4409	0.1791	0.5459	0.019*	
C72	0.33769 (13)	0.16760 (15)	0.79961 (13)	0.0206 (5)	
H72	0.3247	0.1646	0.7479	0.025*	
C53	0.43128 (11)	0.07298 (14)	0.59626 (13)	0.0143 (5)	
C56	0.25310 (13)	0.24601 (15)	0.56476 (14)	0.0198 (5)	
H56	0.2216	0.2173	0.5285	0.024*	
C10	0.97019 (15)	0.25843 (18)	0.54138 (15)	0.0308 (6)	
H10	0.9658	0.2812	0.5863	0.037*	
C3S	0.7913 (2)	0.3101 (2)	0.4286 (2)	0.0613 (11)	
H3S1	0.7927	0.2979	0.4798	0.092*	
H3S2	0.7811	0.3663	0.4195	0.092*	
H3S3	0.8362	0.2978	0.4177	0.092*	
C18	0.99601 (12)	−0.01346 (14)	0.31244 (13)	0.0180 (5)	
H18	1.0412	0.001	0.3084	0.022*	
C64	0.45917 (12)	0.20529 (13)	0.79421 (13)	0.0147 (5)	
C48	0.42152 (11)	−0.00937 (14)	0.58107 (13)	0.0155 (5)	
C34	1.17054 (14)	0.05419 (16)	0.47784 (15)	0.0262 (6)	
H34	1.1644	0.0295	0.5213	0.031*	
C44	0.43700 (11)	0.09951 (13)	0.67017 (12)	0.0138 (5)	
C22	0.79677 (13)	−0.08122 (15)	0.32964 (14)	0.0213 (5)	
H22	0.7855	−0.1357	0.3302	0.026*	
C20	0.91353 (13)	−0.11548 (15)	0.31596 (14)	0.0219 (5)	
H20	0.9019	−0.1699	0.3152	0.026*	
C68	0.42456 (13)	0.18990 (14)	0.91148 (13)	0.0174 (5)	
C27	1.06450 (12)	0.14510 (14)	0.30637 (13)	0.0169 (5)	
C55	0.31566 (12)	0.21079 (14)	0.60119 (13)	0.0162 (5)	
H55	0.326	0.1586	0.5885	0.019*	
C51	0.42607 (12)	0.09599 (15)	0.46746 (13)	0.0187 (5)	
H51	0.4286	0.1313	0.4289	0.022*	
C45	0.43799 (12)	0.04421 (14)	0.72423 (13)	0.0158 (5)	
H45	0.444	0.0614	0.7733	0.019*	
C7	0.97909 (12)	0.19186 (15)	0.40698 (13)	0.0187 (5)	
C62	0.39025 (13)	0.37353 (15)	0.72987 (13)	0.0198 (5)	
H62	0.4324	0.3512	0.756	0.024*	
C25	0.82850 (13)	0.07842 (15)	0.32955 (14)	0.0205 (5)	
H25	0.8385	0.1333	0.3297	0.025*	
C15	1.04497 (14)	0.31208 (16)	0.38265 (16)	0.0283 (2)	
H15	1.0497	0.2912	0.3371	0.034*	
C61	0.37271 (14)	0.44802 (15)	0.74651 (14)	0.0253 (6)	
H61	0.4027	0.4767	0.7841	0.03*	
C65	0.52473 (13)	0.22282 (13)	0.83309 (13)	0.0172 (5)	
H65	0.5593	0.2351	0.8072	0.021*	
C57	0.23786 (13)	0.32114 (15)	0.58155 (14)	0.0215 (5)	
H57	0.196	0.3448	0.5563	0.026*	
C54	0.36217 (12)	0.25042 (14)	0.65473 (12)	0.0145 (5)	
C21	0.86374 (13)	−0.05871 (15)	0.32365 (13)	0.0191 (5)	
C40	0.56711 (14)	0.44663 (15)	0.59914 (14)	0.0253 (6)	
H40	0.5769	0.4987	0.5857	0.03*	
C35	1.11436 (13)	0.08241 (15)	0.42834 (14)	0.0215 (5)	
H35	1.0698	0.0781	0.4385	0.026*	
C58	0.28366 (13)	0.36425 (15)	0.63605 (14)	0.0207 (5)	
C71	0.28949 (14)	0.15189 (16)	0.83948 (14)	0.0253 (6)	
H71	0.2437	0.1387	0.8152	0.03*	
C26	0.88095 (12)	0.02320 (14)	0.32458 (13)	0.0172 (5)	
C32	1.24612 (14)	0.09633 (16)	0.40218 (16)	0.0289 (6)	
H32	1.2914	0.1019	0.3941	0.035*	
C70	0.30738 (15)	0.15521 (16)	0.91627 (15)	0.0276 (6)	
H70	0.2736	0.1448	0.9437	0.033*	
C30	1.19871 (13)	0.15532 (15)	0.28205 (16)	0.0258 (6)	
H30	1.2441	0.1606	0.2741	0.031*	
C3	0.82824 (13)	0.39243 (15)	0.19905 (15)	0.0245 (6)	
H3	0.8103	0.4444	0.1901	0.029*	
C17	0.94997 (12)	0.04497 (14)	0.32094 (13)	0.0159 (5)	
C69	0.37318 (14)	0.17335 (15)	0.95109 (14)	0.0232 (6)	
H69	0.385	0.175	1.0029	0.028*	
C46	0.43032 (12)	−0.03718 (14)	0.70854 (13)	0.0179 (5)	
H46	0.4317	−0.074	0.747	0.022*	
C12	1.03281 (14)	0.37690 (16)	0.51546 (16)	0.0283 (2)	
H12	1.0285	0.3996	0.5604	0.034*	
C28	1.07644 (13)	0.17322 (14)	0.24157 (14)	0.0211 (5)	
H28	1.0384	0.1901	0.2045	0.025*	
C24	0.76388 (13)	0.05461 (15)	0.33411 (14)	0.0230 (5)	
H24	0.7296	0.0928	0.3369	0.028*	
C1	0.89609 (14)	0.12360 (16)	0.17148 (14)	0.0249 (6)	
C36	1.12161 (12)	0.11787 (14)	0.36217 (14)	0.0185 (5)	
C31	1.18918 (13)	0.12442 (15)	0.34891 (15)	0.0223 (5)	
C4	0.87534 (13)	0.37951 (15)	0.26451 (14)	0.0215 (5)	
C19	0.97786 (13)	−0.09378 (15)	0.30967 (14)	0.0209 (5)	
H19	1.0104	−0.1328	0.3034	0.025*	
C47	0.42097 (12)	−0.06313 (14)	0.63843 (13)	0.0180 (5)	
H47	0.414	−0.1178	0.628	0.022*	
C42	0.44669 (15)	0.49561 (16)	0.59086 (15)	0.0288 (6)	
H42A	0.4047	0.4754	0.5583	0.043*	
H42B	0.4645	0.5404	0.5676	0.043*	
H42C	0.4359	0.5129	0.6368	0.043*	
C2	0.80491 (13)	0.33581 (16)	0.14513 (15)	0.0249 (6)	
C66	0.54236 (13)	0.22314 (14)	0.91011 (13)	0.0196 (5)	
H66	0.5883	0.2345	0.9353	0.024*	
C49	0.41249 (12)	−0.03627 (15)	0.50793 (13)	0.0181 (5)	
H49	0.405	−0.0909	0.4975	0.022*	
C73	0.40621 (13)	0.18809 (13)	0.83337 (13)	0.0165 (5)	
C16	1.00927 (14)	0.26749 (16)	0.42584 (16)	0.0283 (2)	
C23	0.74805 (13)	−0.02628 (16)	0.33467 (14)	0.0229 (6)	
H23	0.7033	−0.0426	0.3386	0.027*	
C8	0.94860 (13)	0.15189 (16)	0.45579 (14)	0.0228 (5)	
H8	0.9305	0.1005	0.4436	0.027*	
C14	1.07306 (14)	0.38447 (16)	0.40426 (15)	0.0283 (2)	
H14	1.0972	0.4126	0.374	0.034*	
C63	0.34676 (12)	0.32891 (14)	0.67429 (13)	0.0175 (5)	
C29	1.14352 (14)	0.17767 (15)	0.22877 (15)	0.0249 (6)	
H29	1.1503	0.1962	0.1832	0.03*	
C67	0.49302 (13)	0.20703 (14)	0.94805 (13)	0.0199 (5)	
H67	0.5049	0.2073	0.9999	0.024*	
C59	0.26762 (14)	0.44216 (16)	0.65436 (15)	0.0267 (6)	
H59	0.2262	0.4663	0.6284	0.032*	
C60	0.31056 (15)	0.48312 (16)	0.70851 (16)	0.0293 (6)	
H60	0.2988	0.535	0.7206	0.035*	
C6	0.89517 (15)	0.44618 (16)	0.31812 (16)	0.0283 (6)	
H6A	0.8835	0.432	0.3645	0.042*	
H6B	0.87	0.494	0.2986	0.042*	
H6C	0.9449	0.4558	0.3262	0.042*	
C39	0.50023 (14)	0.43147 (15)	0.60603 (13)	0.0221 (5)	
C9	0.94373 (14)	0.18528 (18)	0.52279 (15)	0.0288 (6)	
H9	0.922	0.1569	0.555	0.035*	
C5	0.74708 (16)	0.35524 (19)	0.08127 (17)	0.0391 (7)	
H5A	0.7645	0.3553	0.0365	0.059*	
H5B	0.7285	0.4075	0.0884	0.059*	
H5C	0.7105	0.3156	0.0772	0.059*	
C41	0.62120 (14)	0.39225 (16)	0.61022 (15)	0.0268 (6)	
C13	1.06635 (14)	0.41745 (16)	0.47135 (15)	0.0283 (2)	
H13	1.0853	0.468	0.4857	0.034*	
C33	1.23727 (14)	0.06142 (17)	0.46478 (15)	0.0300 (6)	
H33	1.276	0.042	0.4995	0.036*	
C43	0.69125 (17)	0.4173 (2)	0.5994 (2)	0.0498 (9)	
H43A	0.7222	0.427	0.647	0.075*	
H43B	0.6865	0.4657	0.5702	0.075*	
H43C	0.7105	0.3753	0.574	0.075*	
C2S	0.73211 (17)	0.2659 (2)	0.3004 (2)	0.0435 (8)	
H2S1	0.6964	0.2295	0.2749	0.065*	
H2S2	0.7768	0.2512	0.2903	0.065*	
H2S3	0.7203	0.3199	0.2833	0.065*	
C1S	0.73633 (15)	0.26151 (17)	0.38060 (19)	0.0367 (7)	

### Atomic displacement parameters (Å^2^)

**Table:**

	*U*^11^	*U*^22^	*U*^33^	*U*^12^	*U*^13^	*U*^23^
Rh2	0.01668 (10)	0.01180 (9)	0.01356 (9)	−0.00149 (7)	0.00425 (7)	0.00001 (6)
Rh1	0.01686 (10)	0.01368 (10)	0.01775 (10)	0.00225 (7)	0.00450 (7)	0.00181 (7)
P2	0.0158 (3)	0.0106 (3)	0.0122 (3)	−0.0007 (2)	0.0036 (2)	−0.0006 (2)
P1	0.0148 (3)	0.0131 (3)	0.0174 (3)	0.0005 (2)	0.0046 (2)	0.0009 (2)
O2	0.0267 (10)	0.0234 (10)	0.0221 (9)	0.0063 (8)	0.0025 (7)	0.0047 (7)
O1	0.0626 (15)	0.0242 (11)	0.0292 (11)	0.0057 (10)	0.0025 (10)	−0.0073 (9)
O4	0.0255 (10)	0.0184 (10)	0.0418 (12)	0.0030 (8)	0.0045 (8)	−0.0021 (8)
O1S	0.0375 (12)	0.0398 (13)	0.0526 (14)	−0.0048 (10)	0.0188 (11)	0.0013 (10)
O3	0.0216 (9)	0.0162 (9)	0.0243 (9)	0.0037 (7)	0.0058 (7)	0.0008 (7)
O5	0.0237 (9)	0.0228 (10)	0.0293 (10)	−0.0043 (7)	0.0114 (8)	0.0035 (8)
O6	0.0246 (9)	0.0131 (8)	0.0178 (9)	−0.0013 (7)	0.0063 (7)	0.0023 (7)
C11	0.0264 (6)	0.0219 (6)	0.0326 (6)	0.0041 (4)	−0.0020 (5)	−0.0034 (5)
C37	0.0202 (13)	0.0176 (13)	0.0206 (13)	−0.0052 (11)	0.0059 (10)	−0.0028 (10)
C50	0.0191 (13)	0.0259 (14)	0.0144 (12)	0.0005 (10)	0.0015 (10)	−0.0068 (10)
C52	0.0150 (12)	0.0149 (12)	0.0183 (12)	0.0002 (9)	0.0042 (9)	0.0000 (9)
C72	0.0254 (13)	0.0217 (13)	0.0153 (12)	−0.0024 (10)	0.0060 (10)	−0.0018 (10)
C53	0.0103 (11)	0.0151 (12)	0.0166 (12)	0.0004 (9)	0.0011 (9)	−0.0013 (9)
C56	0.0179 (12)	0.0212 (13)	0.0210 (13)	−0.0013 (10)	0.0056 (10)	0.0012 (10)
C10	0.0325 (15)	0.0394 (17)	0.0190 (14)	0.0095 (13)	0.0023 (11)	−0.0049 (12)
C3S	0.051 (2)	0.046 (2)	0.080 (3)	−0.0113 (18)	0.000 (2)	0.005 (2)
C18	0.0180 (12)	0.0187 (12)	0.0178 (12)	0.0004 (10)	0.0051 (10)	0.0020 (10)
C64	0.0230 (12)	0.0084 (11)	0.0132 (11)	−0.0005 (9)	0.0051 (9)	−0.0011 (9)
C48	0.0112 (11)	0.0146 (12)	0.0204 (12)	0.0004 (9)	0.0025 (9)	−0.0009 (9)
C34	0.0270 (14)	0.0260 (14)	0.0222 (14)	0.0041 (11)	−0.0017 (11)	−0.0055 (11)
C44	0.0124 (11)	0.0121 (11)	0.0166 (12)	−0.0002 (9)	0.0023 (9)	−0.0022 (9)
C22	0.0256 (13)	0.0175 (13)	0.0207 (13)	−0.0050 (10)	0.0050 (10)	0.0004 (10)
C20	0.0288 (14)	0.0137 (12)	0.0239 (13)	−0.0023 (10)	0.0074 (11)	−0.0004 (10)
C68	0.0272 (13)	0.0098 (11)	0.0163 (12)	0.0030 (10)	0.0075 (10)	−0.0007 (9)
C27	0.0175 (12)	0.0124 (12)	0.0216 (13)	−0.0004 (9)	0.0060 (10)	−0.0006 (9)
C55	0.0182 (12)	0.0143 (12)	0.0173 (12)	0.0003 (9)	0.0065 (9)	0.0007 (9)
C51	0.0161 (12)	0.0237 (13)	0.0154 (12)	0.0003 (10)	0.0019 (9)	0.0012 (10)
C45	0.0145 (12)	0.0166 (12)	0.0167 (12)	0.0006 (9)	0.0040 (9)	−0.0006 (9)
C7	0.0175 (12)	0.0209 (13)	0.0173 (12)	0.0061 (10)	0.0035 (9)	0.0008 (10)
C62	0.0247 (13)	0.0177 (13)	0.0184 (13)	0.0014 (10)	0.0079 (10)	−0.0010 (10)
C25	0.0209 (13)	0.0159 (12)	0.0253 (13)	0.0008 (10)	0.0064 (10)	0.0034 (10)
C15	0.0264 (6)	0.0219 (6)	0.0326 (6)	0.0041 (4)	−0.0020 (5)	−0.0034 (5)
C61	0.0334 (15)	0.0204 (13)	0.0237 (14)	0.0002 (11)	0.0095 (11)	−0.0052 (11)
C65	0.0235 (13)	0.0112 (11)	0.0175 (12)	−0.0006 (9)	0.0062 (10)	−0.0011 (9)
C57	0.0185 (12)	0.0246 (14)	0.0226 (13)	0.0041 (10)	0.0076 (10)	0.0055 (10)
C54	0.0187 (12)	0.0141 (11)	0.0120 (11)	−0.0004 (9)	0.0061 (9)	0.0026 (9)
C21	0.0208 (12)	0.0198 (13)	0.0165 (12)	−0.0019 (10)	0.0038 (10)	0.0009 (10)
C40	0.0393 (16)	0.0168 (13)	0.0225 (14)	−0.0074 (11)	0.0127 (12)	0.0016 (10)
C35	0.0199 (13)	0.0217 (13)	0.0218 (13)	0.0013 (10)	0.0023 (10)	−0.0046 (10)
C58	0.0233 (13)	0.0185 (13)	0.0228 (13)	0.0034 (10)	0.0105 (10)	0.0027 (10)
C71	0.0249 (14)	0.0293 (15)	0.0240 (14)	−0.0037 (11)	0.0101 (11)	−0.0024 (11)
C26	0.0174 (12)	0.0193 (12)	0.0142 (12)	−0.0005 (10)	0.0022 (9)	0.0031 (9)
C32	0.0170 (13)	0.0281 (15)	0.0399 (16)	0.0000 (11)	0.0027 (11)	−0.0127 (12)
C70	0.0339 (15)	0.0284 (15)	0.0258 (14)	−0.0035 (12)	0.0181 (12)	−0.0013 (11)
C30	0.0193 (13)	0.0185 (13)	0.0432 (16)	−0.0013 (10)	0.0146 (12)	−0.0034 (12)
C3	0.0260 (14)	0.0164 (13)	0.0338 (15)	0.0056 (11)	0.0120 (11)	0.0068 (11)
C17	0.0189 (12)	0.0144 (12)	0.0147 (12)	−0.0005 (9)	0.0040 (9)	0.0001 (9)
C69	0.0345 (15)	0.0218 (13)	0.0158 (12)	0.0006 (11)	0.0110 (11)	−0.0037 (10)
C46	0.0199 (12)	0.0140 (12)	0.0201 (12)	0.0011 (10)	0.0049 (10)	0.0038 (10)
C12	0.0264 (6)	0.0219 (6)	0.0326 (6)	0.0041 (4)	−0.0020 (5)	−0.0034 (5)
C28	0.0238 (13)	0.0136 (12)	0.0271 (14)	0.0024 (10)	0.0085 (11)	0.0028 (10)
C24	0.0184 (13)	0.0235 (13)	0.0272 (14)	0.0037 (10)	0.0052 (11)	0.0028 (11)
C1	0.0273 (14)	0.0235 (14)	0.0220 (13)	0.0043 (11)	0.0013 (11)	0.0052 (11)
C36	0.0175 (12)	0.0135 (12)	0.0238 (13)	−0.0004 (9)	0.0035 (10)	−0.0055 (10)
C31	0.0193 (13)	0.0170 (13)	0.0306 (14)	−0.0007 (10)	0.0055 (11)	−0.0077 (11)
C4	0.0201 (13)	0.0169 (13)	0.0308 (14)	0.0019 (10)	0.0127 (11)	0.0025 (11)
C19	0.0246 (13)	0.0163 (12)	0.0222 (13)	0.0032 (10)	0.0063 (10)	−0.0003 (10)
C47	0.0149 (12)	0.0127 (12)	0.0258 (13)	−0.0003 (9)	0.0034 (10)	−0.0008 (10)
C42	0.0420 (17)	0.0197 (14)	0.0247 (14)	0.0031 (12)	0.0077 (12)	0.0033 (11)
C2	0.0245 (14)	0.0239 (14)	0.0271 (14)	0.0067 (11)	0.0073 (11)	0.0084 (11)
C66	0.0245 (13)	0.0135 (12)	0.0186 (13)	0.0015 (10)	−0.0001 (10)	−0.0007 (10)
C49	0.0154 (12)	0.0161 (12)	0.0210 (13)	0.0003 (9)	0.0007 (10)	−0.0066 (10)
C73	0.0236 (13)	0.0097 (11)	0.0170 (12)	0.0018 (9)	0.0064 (10)	0.0005 (9)
C16	0.0264 (6)	0.0219 (6)	0.0326 (6)	0.0041 (4)	−0.0020 (5)	−0.0034 (5)
C23	0.0174 (12)	0.0288 (14)	0.0223 (13)	−0.0038 (11)	0.0042 (10)	0.0036 (11)
C8	0.0203 (13)	0.0258 (14)	0.0219 (13)	0.0020 (11)	0.0041 (10)	−0.0004 (11)
C14	0.0264 (6)	0.0219 (6)	0.0326 (6)	0.0041 (4)	−0.0020 (5)	−0.0034 (5)
C63	0.0205 (12)	0.0172 (12)	0.0175 (12)	0.0012 (10)	0.0099 (10)	0.0021 (10)
C29	0.0280 (14)	0.0190 (13)	0.0325 (15)	0.0022 (11)	0.0169 (12)	0.0030 (11)
C67	0.0316 (14)	0.0138 (12)	0.0136 (12)	0.0032 (10)	0.0033 (10)	−0.0003 (9)
C59	0.0264 (14)	0.0228 (14)	0.0326 (15)	0.0088 (11)	0.0100 (11)	0.0015 (11)
C60	0.0360 (16)	0.0179 (13)	0.0365 (16)	0.0057 (12)	0.0135 (13)	−0.0035 (12)
C6	0.0340 (15)	0.0192 (14)	0.0343 (16)	0.0053 (11)	0.0131 (12)	0.0004 (11)
C39	0.0371 (15)	0.0170 (13)	0.0122 (12)	−0.0020 (11)	0.0052 (10)	−0.0001 (10)
C9	0.0291 (15)	0.0376 (16)	0.0207 (14)	0.0041 (12)	0.0078 (11)	0.0031 (12)
C5	0.0415 (18)	0.0333 (17)	0.0362 (17)	0.0142 (14)	−0.0046 (13)	0.0048 (13)
C41	0.0331 (15)	0.0257 (15)	0.0244 (14)	−0.0103 (12)	0.0127 (11)	0.0007 (11)
C13	0.0264 (6)	0.0219 (6)	0.0326 (6)	0.0041 (4)	−0.0020 (5)	−0.0034 (5)
C33	0.0237 (14)	0.0321 (16)	0.0283 (15)	0.0064 (12)	−0.0067 (11)	−0.0101 (12)
C43	0.0402 (19)	0.0377 (19)	0.079 (3)	−0.0101 (15)	0.0301 (18)	0.0112 (18)
C2S	0.0336 (17)	0.0380 (18)	0.061 (2)	0.0113 (14)	0.0142 (15)	0.0200 (16)
C1S	0.0233 (15)	0.0247 (15)	0.062 (2)	0.0047 (12)	0.0091 (14)	0.0078 (14)

### Geometric parameters (Å, º)

**Table:**

Rh2—C37	1.801 (3)	C15—C16	1.411 (4)
Rh2—O6	2.0374 (16)	C15—H15	0.95
Rh2—O5	2.0649 (17)	C61—C60	1.413 (4)
Rh2—P2	2.2588 (6)	C61—H61	0.95
Rh1—C1	1.817 (3)	C65—C66	1.412 (3)
Rh1—O3	2.0380 (17)	C65—H65	0.95
Rh1—O2	2.0633 (17)	C57—C58	1.412 (4)
Rh1—P1	2.2513 (6)	C57—H57	0.95
P2—C44	1.842 (2)	C54—C63	1.431 (3)
P2—C64	1.846 (2)	C21—C26	1.428 (3)
P2—C54	1.850 (2)	C40—C39	1.388 (4)
P1—C27	1.835 (2)	C40—C41	1.396 (4)
P1—C7	1.839 (2)	C40—H40	0.95
P1—C17	1.841 (2)	C35—C36	1.418 (4)
O2—C2	1.274 (3)	C35—H35	0.95
O1—C1	1.144 (3)	C58—C59	1.418 (4)
O4—C37	1.152 (3)	C58—C63	1.430 (3)
O1S—C1S	1.215 (4)	C71—C70	1.409 (4)
O3—C4	1.281 (3)	C71—H71	0.95
O5—C41	1.274 (3)	C26—C17	1.437 (3)
O6—C39	1.285 (3)	C32—C33	1.364 (4)
C11—C10	1.411 (4)	C32—C31	1.416 (4)
C11—C12	1.424 (4)	C32—H32	0.95
C11—C16	1.446 (4)	C70—C69	1.361 (4)
C50—C49	1.359 (4)	C70—H70	0.95
C50—C51	1.409 (4)	C30—C29	1.363 (4)
C50—H50	0.95	C30—C31	1.413 (4)
C52—C51	1.368 (3)	C30—H30	0.95
C52—C53	1.421 (3)	C3—C4	1.387 (4)
C52—H52	0.95	C3—C2	1.397 (4)
C72—C71	1.368 (4)	C3—H3	0.95
C72—C73	1.411 (4)	C69—H69	0.95
C72—H72	0.95	C46—C47	1.362 (4)
C53—C48	1.428 (3)	C46—H46	0.95
C53—C44	1.441 (3)	C12—C13	1.361 (4)
C56—C57	1.361 (4)	C12—H12	0.95
C56—C55	1.411 (3)	C28—C29	1.409 (4)
C56—H56	0.95	C28—H28	0.95
C10—C9	1.361 (4)	C24—C23	1.406 (4)
C10—H10	0.95	C24—H24	0.95
C3S—C1S	1.498 (5)	C36—C31	1.424 (3)
C3S—H3S1	0.98	C4—C6	1.506 (4)
C3S—H3S2	0.98	C19—H19	0.95
C3S—H3S3	0.98	C47—H47	0.95
C18—C17	1.381 (3)	C42—C39	1.503 (4)
C18—C19	1.405 (3)	C42—H42A	0.98
C18—H18	0.95	C42—H42B	0.98
C64—C65	1.377 (3)	C42—H42C	0.98
C64—C73	1.443 (3)	C2—C5	1.502 (4)
C48—C47	1.414 (3)	C66—C67	1.363 (4)
C48—C49	1.422 (3)	C66—H66	0.95
C34—C35	1.370 (4)	C49—H49	0.95
C34—C33	1.407 (4)	C23—H23	0.95
C34—H34	0.95	C8—C9	1.404 (4)
C44—C45	1.379 (3)	C8—H8	0.95
C22—C23	1.361 (4)	C14—C13	1.413 (4)
C22—C21	1.413 (4)	C14—H14	0.95
C22—H22	0.95	C29—H29	0.95
C20—C19	1.361 (4)	C67—H67	0.95
C20—C21	1.410 (4)	C59—C60	1.363 (4)
C20—H20	0.95	C59—H59	0.95
C68—C67	1.411 (4)	C60—H60	0.95
C68—C69	1.420 (4)	C6—H6A	0.98
C68—C73	1.433 (3)	C6—H6B	0.98
C27—C28	1.377 (4)	C6—H6C	0.98
C27—C36	1.439 (3)	C9—H9	0.95
C55—C54	1.380 (3)	C5—H5A	0.98
C55—H55	0.95	C5—H5B	0.98
C51—H51	0.95	C5—H5C	0.98
C45—C46	1.411 (3)	C41—C43	1.512 (4)
C45—H45	0.95	C13—H13	0.95
C7—C8	1.385 (4)	C33—H33	0.95
C7—C16	1.425 (4)	C43—H43A	0.98
C62—C61	1.364 (4)	C43—H43B	0.98
C62—C63	1.417 (4)	C43—H43C	0.98
C62—H62	0.95	C2S—C1S	1.494 (5)
C25—C24	1.367 (4)	C2S—H2S1	0.98
C25—C26	1.419 (3)	C2S—H2S2	0.98
C25—H25	0.95	C2S—H2S3	0.98
C15—C14	1.370 (4)		
			
C37—Rh2—O6	175.48 (9)	C33—C32—C31	121.3 (3)
C37—Rh2—O5	90.28 (9)	C33—C32—H32	119.3
O6—Rh2—O5	88.58 (7)	C31—C32—H32	119.3
C37—Rh2—P2	88.18 (8)	C69—C70—C71	119.8 (2)
O6—Rh2—P2	93.22 (5)	C69—C70—H70	120.1
O5—Rh2—P2	176.14 (5)	C71—C70—H70	120.1
C1—Rh1—O3	178.85 (10)	C29—C30—C31	120.7 (2)
C1—Rh1—O2	90.07 (9)	C29—C30—H30	119.6
O3—Rh1—O2	88.78 (7)	C31—C30—H30	119.6
C1—Rh1—P1	88.35 (8)	C4—C3—C2	125.8 (2)
O3—Rh1—P1	92.78 (5)	C4—C3—H3	117.1
O2—Rh1—P1	176.45 (5)	C2—C3—H3	117.1
C44—P2—C64	103.47 (10)	C18—C17—C26	119.1 (2)
C44—P2—C54	105.48 (10)	C18—C17—P1	119.32 (18)
C64—P2—C54	107.83 (11)	C26—C17—P1	121.17 (18)
C44—P2—Rh2	114.33 (8)	C70—C69—C68	121.3 (2)
C64—P2—Rh2	112.65 (8)	C70—C69—H69	119.4
C54—P2—Rh2	112.38 (7)	C68—C69—H69	119.4
C27—P1—C7	105.28 (11)	C47—C46—C45	120.2 (2)
C27—P1—C17	104.22 (11)	C47—C46—H46	119.9
C7—P1—C17	106.77 (11)	C45—C46—H46	119.9
C27—P1—Rh1	115.36 (8)	C13—C12—C11	120.9 (3)
C7—P1—Rh1	113.44 (8)	C13—C12—H12	119.5
C17—P1—Rh1	110.99 (8)	C11—C12—H12	119.5
C2—O2—Rh1	126.44 (17)	C27—C28—C29	121.8 (2)
C4—O3—Rh1	126.77 (16)	C27—C28—H28	119.1
C41—O5—Rh2	126.54 (17)	C29—C28—H28	119.1
C39—O6—Rh2	127.15 (16)	C25—C24—C23	120.2 (2)
C10—C11—C12	120.9 (3)	C25—C24—H24	119.9
C10—C11—C16	119.7 (3)	C23—C24—H24	119.9
C12—C11—C16	119.4 (3)	O1—C1—Rh1	177.6 (2)
O4—C37—Rh2	176.4 (2)	C35—C36—C31	118.1 (2)
C49—C50—C51	120.2 (2)	C35—C36—C27	123.9 (2)
C49—C50—H50	119.9	C31—C36—C27	117.9 (2)
C51—C50—H50	119.9	C30—C31—C32	120.6 (2)
C51—C52—C53	121.4 (2)	C30—C31—C36	120.2 (2)
C51—C52—H52	119.3	C32—C31—C36	119.1 (2)
C53—C52—H52	119.3	O3—C4—C3	125.9 (2)
C71—C72—C73	121.7 (2)	O3—C4—C6	114.2 (2)
C71—C72—H72	119.2	C3—C4—C6	120.0 (2)
C73—C72—H72	119.2	C20—C19—C18	119.8 (2)
C52—C53—C48	117.7 (2)	C20—C19—H19	120.1
C52—C53—C44	123.9 (2)	C18—C19—H19	120.1
C48—C53—C44	118.4 (2)	C46—C47—C48	120.5 (2)
C57—C56—C55	120.0 (2)	C46—C47—H47	119.7
C57—C56—H56	120	C48—C47—H47	119.7
C55—C56—H56	120	C39—C42—H42A	109.5
C9—C10—C11	121.1 (3)	C39—C42—H42B	109.5
C9—C10—H10	119.4	H42A—C42—H42B	109.5
C11—C10—H10	119.4	C39—C42—H42C	109.5
C1S—C3S—H3S1	109.5	H42A—C42—H42C	109.5
C1S—C3S—H3S2	109.5	H42B—C42—H42C	109.5
H3S1—C3S—H3S2	109.5	O2—C2—C3	125.6 (2)
C1S—C3S—H3S3	109.5	O2—C2—C5	114.4 (2)
H3S1—C3S—H3S3	109.5	C3—C2—C5	120.0 (2)
H3S2—C3S—H3S3	109.5	C67—C66—C65	119.6 (2)
C17—C18—C19	121.8 (2)	C67—C66—H66	120.2
C17—C18—H18	119.1	C65—C66—H66	120.2
C19—C18—H18	119.1	C50—C49—C48	121.0 (2)
C65—C64—C73	119.0 (2)	C50—C49—H49	119.5
C65—C64—P2	115.81 (18)	C48—C49—H49	119.5
C73—C64—P2	125.21 (18)	C72—C73—C68	117.7 (2)
C47—C48—C49	120.7 (2)	C72—C73—C64	124.1 (2)
C47—C48—C53	120.0 (2)	C68—C73—C64	118.1 (2)
C49—C48—C53	119.2 (2)	C15—C16—C7	125.1 (3)
C35—C34—C33	120.5 (3)	C15—C16—C11	117.1 (2)
C35—C34—H34	119.7	C7—C16—C11	117.8 (3)
C33—C34—H34	119.7	C22—C23—C24	120.1 (2)
C45—C44—C53	119.0 (2)	C22—C23—H23	119.9
C45—C44—P2	119.77 (18)	C24—C23—H23	119.9
C53—C44—P2	121.19 (17)	C7—C8—C9	121.8 (3)
C23—C22—C21	121.2 (2)	C7—C8—H8	119.1
C23—C22—H22	119.4	C9—C8—H8	119.1
C21—C22—H22	119.4	C15—C14—C13	120.3 (3)
C19—C20—C21	121.3 (2)	C15—C14—H14	119.8
C19—C20—H20	119.4	C13—C14—H14	119.8
C21—C20—H20	119.4	C62—C63—C58	118.0 (2)
C67—C68—C69	120.9 (2)	C62—C63—C54	123.7 (2)
C67—C68—C73	120.1 (2)	C58—C63—C54	118.3 (2)
C69—C68—C73	119.0 (2)	C30—C29—C28	119.8 (2)
C28—C27—C36	119.5 (2)	C30—C29—H29	120.1
C28—C27—P1	116.04 (18)	C28—C29—H29	120.1
C36—C27—P1	124.42 (18)	C66—C67—C68	121.0 (2)
C54—C55—C56	121.5 (2)	C66—C67—H67	119.5
C54—C55—H55	119.2	C68—C67—H67	119.5
C56—C55—H55	119.2	C60—C59—C58	121.2 (3)
C52—C51—C50	120.3 (2)	C60—C59—H59	119.4
C52—C51—H51	119.8	C58—C59—H59	119.4
C50—C51—H51	119.8	C59—C60—C61	119.6 (2)
C44—C45—C46	121.8 (2)	C59—C60—H60	120.2
C44—C45—H45	119.1	C61—C60—H60	120.2
C46—C45—H45	119.1	C4—C6—H6A	109.5
C8—C7—C16	119.8 (2)	C4—C6—H6B	109.5
C8—C7—P1	119.18 (19)	H6A—C6—H6B	109.5
C16—C7—P1	120.9 (2)	C4—C6—H6C	109.5
C61—C62—C63	121.2 (2)	H6A—C6—H6C	109.5
C61—C62—H62	119.4	H6B—C6—H6C	109.5
C63—C62—H62	119.4	O6—C39—C40	125.7 (2)
C24—C25—C26	121.6 (2)	O6—C39—C42	114.2 (2)
C24—C25—H25	119.2	C40—C39—C42	120.1 (2)
C26—C25—H25	119.2	C10—C9—C8	119.8 (3)
C14—C15—C16	122.1 (3)	C10—C9—H9	120.1
C14—C15—H15	118.9	C8—C9—H9	120.1
C16—C15—H15	118.9	C2—C5—H5A	109.5
C62—C61—C60	120.8 (3)	C2—C5—H5B	109.5
C62—C61—H61	119.6	H5A—C5—H5B	109.5
C60—C61—H61	119.6	C2—C5—H5C	109.5
C64—C65—C66	122.3 (2)	H5A—C5—H5C	109.5
C64—C65—H65	118.9	H5B—C5—H5C	109.5
C66—C65—H65	118.9	O5—C41—C40	125.7 (2)
C56—C57—C58	120.7 (2)	O5—C41—C43	114.5 (3)
C56—C57—H57	119.6	C40—C41—C43	119.8 (3)
C58—C57—H57	119.6	C12—C13—C14	120.1 (3)
C55—C54—C63	119.5 (2)	C12—C13—H13	119.9
C55—C54—P2	119.44 (18)	C14—C13—H13	119.9
C63—C54—P2	120.80 (18)	C32—C33—C34	119.8 (3)
C20—C21—C22	121.3 (2)	C32—C33—H33	120.1
C20—C21—C26	119.5 (2)	C34—C33—H33	120.1
C22—C21—C26	119.2 (2)	C41—C43—H43A	109.5
C39—C40—C41	125.9 (2)	C41—C43—H43B	109.5
C39—C40—H40	117.1	H43A—C43—H43B	109.5
C41—C40—H40	117.1	C41—C43—H43C	109.5
C34—C35—C36	121.1 (2)	H43A—C43—H43C	109.5
C34—C35—H35	119.4	H43B—C43—H43C	109.5
C36—C35—H35	119.4	C1S—C2S—H2S1	109.5
C57—C58—C59	120.9 (2)	C1S—C2S—H2S2	109.5
C57—C58—C63	119.9 (2)	H2S1—C2S—H2S2	109.5
C59—C58—C63	119.1 (2)	C1S—C2S—H2S3	109.5
C72—C71—C70	120.4 (2)	H2S1—C2S—H2S3	109.5
C72—C71—H71	119.8	H2S2—C2S—H2S3	109.5
C70—C71—H71	119.8	O1S—C1S—C2S	121.2 (3)
C25—C26—C21	117.6 (2)	O1S—C1S—C3S	122.1 (3)
C25—C26—C17	123.9 (2)	C2S—C1S—C3S	116.7 (3)
C21—C26—C17	118.5 (2)		
			
C37—Rh2—P2—C44	−36.07 (11)	Rh1—P1—C17—C26	−51.6 (2)
O6—Rh2—P2—C44	139.63 (9)	C71—C70—C69—C68	0.6 (4)
C37—Rh2—P2—C64	81.68 (11)	C67—C68—C69—C70	−178.7 (2)
O6—Rh2—P2—C64	−102.61 (9)	C73—C68—C69—C70	0.6 (4)
C37—Rh2—P2—C54	−156.28 (11)	C44—C45—C46—C47	−0.6 (4)
O6—Rh2—P2—C54	19.43 (9)	C10—C11—C12—C13	179.1 (3)
C1—Rh1—P1—C27	80.66 (12)	C16—C11—C12—C13	−1.5 (4)
O3—Rh1—P1—C27	−99.52 (10)	C36—C27—C28—C29	−1.3 (4)
C1—Rh1—P1—C7	−157.77 (12)	P1—C27—C28—C29	176.3 (2)
O3—Rh1—P1—C7	22.04 (10)	C26—C25—C24—C23	0.6 (4)
C1—Rh1—P1—C17	−37.57 (12)	C34—C35—C36—C31	−0.9 (4)
O3—Rh1—P1—C17	142.25 (9)	C34—C35—C36—C27	176.8 (2)
C1—Rh1—O2—C2	−179.8 (2)	C28—C27—C36—C35	−175.0 (2)
O3—Rh1—O2—C2	0.3 (2)	P1—C27—C36—C35	7.6 (3)
O2—Rh1—O3—C4	−7.33 (19)	C28—C27—C36—C31	2.6 (3)
P1—Rh1—O3—C4	175.86 (19)	P1—C27—C36—C31	−174.75 (18)
C37—Rh2—O5—C41	−177.5 (2)	C29—C30—C31—C32	175.7 (2)
O6—Rh2—O5—C41	6.9 (2)	C29—C30—C31—C36	−1.4 (4)
O5—Rh2—O6—C39	−5.34 (19)	C33—C32—C31—C30	−175.5 (3)
P2—Rh2—O6—C39	171.24 (18)	C33—C32—C31—C36	1.7 (4)
C51—C52—C53—C48	1.0 (3)	C35—C36—C31—C30	176.5 (2)
C51—C52—C53—C44	−178.1 (2)	C27—C36—C31—C30	−1.3 (3)
C12—C11—C10—C9	−178.9 (3)	C35—C36—C31—C32	−0.7 (3)
C16—C11—C10—C9	1.8 (4)	C27—C36—C31—C32	−178.5 (2)
C44—P2—C64—C65	108.30 (18)	Rh1—O3—C4—C3	8.3 (4)
C54—P2—C64—C65	−140.27 (18)	Rh1—O3—C4—C6	−172.57 (16)
Rh2—P2—C64—C65	−15.7 (2)	C2—C3—C4—O3	−0.2 (4)
C44—P2—C64—C73	−71.1 (2)	C2—C3—C4—C6	−179.2 (2)
C54—P2—C64—C73	40.3 (2)	C21—C20—C19—C18	1.4 (4)
Rh2—P2—C64—C73	164.92 (17)	C17—C18—C19—C20	−0.5 (4)
C52—C53—C48—C47	177.9 (2)	C45—C46—C47—C48	2.5 (4)
C44—C53—C48—C47	−3.0 (3)	C49—C48—C47—C46	179.3 (2)
C52—C53—C48—C49	−2.1 (3)	C53—C48—C47—C46	−0.6 (3)
C44—C53—C48—C49	177.0 (2)	Rh1—O2—C2—C3	6.3 (4)
C52—C53—C44—C45	−176.2 (2)	Rh1—O2—C2—C5	−173.66 (19)
C48—C53—C44—C45	4.7 (3)	C4—C3—C2—O2	−7.8 (4)
C52—C53—C44—P2	0.9 (3)	C4—C3—C2—C5	172.1 (3)
C48—C53—C44—P2	−178.12 (16)	C64—C65—C66—C67	−1.2 (4)
C64—P2—C44—C45	1.7 (2)	C51—C50—C49—C48	0.6 (4)
C54—P2—C44—C45	−111.41 (19)	C47—C48—C49—C50	−178.6 (2)
Rh2—P2—C44—C45	124.61 (17)	C53—C48—C49—C50	1.3 (3)
C64—P2—C44—C53	−175.38 (18)	C71—C72—C73—C68	1.7 (4)
C54—P2—C44—C53	71.5 (2)	C71—C72—C73—C64	−180.0 (2)
Rh2—P2—C44—C53	−52.5 (2)	C67—C68—C73—C72	177.6 (2)
C7—P1—C27—C28	−132.80 (19)	C69—C68—C73—C72	−1.7 (3)
C17—P1—C27—C28	115.01 (19)	C67—C68—C73—C64	−0.9 (3)
Rh1—P1—C27—C28	−6.9 (2)	C69—C68—C73—C64	179.8 (2)
C7—P1—C27—C36	44.7 (2)	C65—C64—C73—C72	−178.5 (2)
C17—P1—C27—C36	−67.5 (2)	P2—C64—C73—C72	0.9 (3)
Rh1—P1—C27—C36	170.53 (17)	C65—C64—C73—C68	−0.2 (3)
C57—C56—C55—C54	0.8 (4)	P2—C64—C73—C68	179.21 (17)
C53—C52—C51—C50	0.9 (4)	C14—C15—C16—C7	−179.7 (3)
C49—C50—C51—C52	−1.7 (4)	C14—C15—C16—C11	−0.8 (4)
C53—C44—C45—C46	−3.0 (3)	C8—C7—C16—C15	176.4 (2)
P2—C44—C45—C46	179.81 (18)	P1—C7—C16—C15	−7.7 (4)
C27—P1—C7—C8	−126.3 (2)	C8—C7—C16—C11	−2.5 (4)
C17—P1—C7—C8	−15.9 (2)	P1—C7—C16—C11	173.38 (19)
Rh1—P1—C7—C8	106.65 (19)	C10—C11—C16—C15	−178.8 (2)
C27—P1—C7—C16	57.8 (2)	C12—C11—C16—C15	1.9 (4)
C17—P1—C7—C16	168.2 (2)	C10—C11—C16—C7	0.2 (4)
Rh1—P1—C7—C16	−69.2 (2)	C12—C11—C16—C7	−179.1 (2)
C63—C62—C61—C60	−0.2 (4)	C21—C22—C23—C24	−0.1 (4)
C73—C64—C65—C66	1.2 (3)	C25—C24—C23—C22	−0.9 (4)
P2—C64—C65—C66	−178.21 (18)	C16—C7—C8—C9	2.8 (4)
C55—C56—C57—C58	−0.9 (4)	P1—C7—C8—C9	−173.1 (2)
C56—C55—C54—C63	0.3 (3)	C16—C15—C14—C13	−0.6 (4)
C56—C55—C54—P2	−173.95 (18)	C61—C62—C63—C58	−0.1 (4)
C44—P2—C54—C55	−17.9 (2)	C61—C62—C63—C54	−179.6 (2)
C64—P2—C54—C55	−127.92 (19)	C57—C58—C63—C62	−178.3 (2)
Rh2—P2—C54—C55	107.34 (18)	C59—C58—C63—C62	0.8 (3)
C44—P2—C54—C63	167.93 (18)	C57—C58—C63—C54	1.2 (3)
C64—P2—C54—C63	57.9 (2)	C59—C58—C63—C54	−179.7 (2)
Rh2—P2—C54—C63	−66.87 (19)	C55—C54—C63—C62	178.2 (2)
C19—C20—C21—C22	179.5 (2)	P2—C54—C63—C62	−7.6 (3)
C19—C20—C21—C26	0.3 (4)	C55—C54—C63—C58	−1.3 (3)
C23—C22—C21—C20	−177.8 (2)	P2—C54—C63—C58	172.89 (17)
C23—C22—C21—C26	1.4 (4)	C31—C30—C29—C28	2.8 (4)
C33—C34—C35—C36	1.4 (4)	C27—C28—C29—C30	−1.4 (4)
C56—C57—C58—C59	−179.2 (2)	C65—C66—C67—C68	0.1 (4)
C56—C57—C58—C63	−0.1 (4)	C69—C68—C67—C66	−179.8 (2)
C73—C72—C71—C70	−0.5 (4)	C73—C68—C67—C66	0.9 (4)
C24—C25—C26—C21	0.7 (4)	C57—C58—C59—C60	177.9 (3)
C24—C25—C26—C17	−178.9 (2)	C63—C58—C59—C60	−1.3 (4)
C20—C21—C26—C25	177.6 (2)	C58—C59—C60—C61	0.9 (4)
C22—C21—C26—C25	−1.7 (3)	C62—C61—C60—C59	−0.2 (4)
C20—C21—C26—C17	−2.8 (3)	Rh2—O6—C39—C40	2.2 (4)
C22—C21—C26—C17	177.9 (2)	Rh2—O6—C39—C42	−176.84 (16)
C72—C71—C70—C69	−0.7 (4)	C41—C40—C39—O6	2.3 (4)
C19—C18—C17—C26	−2.1 (4)	C41—C40—C39—C42	−178.7 (2)
C19—C18—C17—P1	−174.75 (19)	C11—C10—C9—C8	−1.5 (4)
C25—C26—C17—C18	−176.7 (2)	C7—C8—C9—C10	−0.8 (4)
C21—C26—C17—C18	3.7 (3)	Rh2—O5—C41—C40	−5.3 (4)
C25—C26—C17—P1	−4.2 (3)	Rh2—O5—C41—C43	175.1 (2)
C21—C26—C17—P1	176.18 (17)	C39—C40—C41—O5	−0.5 (4)
C27—P1—C17—C18	−3.9 (2)	C39—C40—C41—C43	179.0 (3)
C7—P1—C17—C18	−115.0 (2)	C11—C12—C13—C14	0.1 (4)
Rh1—P1—C17—C18	120.88 (18)	C15—C14—C13—C12	1.0 (4)
C27—P1—C17—C26	−176.36 (19)	C31—C32—C33—C34	−1.2 (4)
C7—P1—C17—C26	72.5 (2)	C35—C34—C33—C32	−0.4 (4)
